# Determining minimal important change for the thyroid-related quality of life questionnaire ThyPRO

**DOI:** 10.1530/EC-21-0026

**Published:** 2021-02-10

**Authors:** Selma Flora Nordqvist, Victor Brun Boesen, Åse Krogh Rasmussen, Ulla Feldt-Rasmussen, Laszlo Hegedüs, Steen Joop Bonnema, Per Karkov Cramon, Torquil Watt, Mogens Groenvold, Jakob Bue Bjorner

**Affiliations:** 1 Department of Medical Endocrinology and Metabolism, Copenhagen University Hospital Rigshospitalet, Copenhagen, Denmark; 2 Institute of Clinical Medicine, University of Copenhagen, Copenhagen, Denmark; 3 Department of Endocrinology and Metabolism, Odense University Hospital, Odense, Denmark; 4 Department of Clinical Physiology and Nuclear Medicine, Bispebjerg and Frederiksberg Hospital, Copenhagen, Denmark; 5 Department of Internal Medicine, Herlev Gentofte Hospital, Copenhagen, Denmark; 6 Department of Public Health, University of Copenhagen, Copenhagen, Denmark; 7 Department of Palliative Medicine, Bispebjerg Hospital, Copenhagen, Denmark; 8 National Research Centre for the Working Environment, Copenhagen, Denmark; 9 QualityMetric Incorporated, LLC, Johnston, Rhode Island, USA

**Keywords:** minimal important change, ThyPRO, thyroid-related quality of life, patient-reported outcomes, interpretability

## Abstract

**Objective:**

ThyPRO is the standard thyroid patient-reported outcome (PRO). The change in scores that patients perceive as important remains to be ascertained. The purpose of this study was to determine values for minimal important change (MIC) for ThyPRO.

**Methods:**

A total of 435 patients treated for benign thyroid diseases completed ThyPRO at baseline and 6 weeks following treatment initiation. At 6 weeks follow-up, patients also completed Global Rating of Change items. For each 0–100 scale, two MIC values were identified: An MIC for groups, using the receiver operating characteristic (ROC) curve method and an MIC for individual patients, using the Reliable Change Index.

**Results:**

ROC analyses provided group-MIC estimates of 6.3–14.3 (score range 0–100). Evaluation of area under the curve (AUC) supported the robustness for 9 of 14 scales (AUC > 0.7). Reliable Change Index estimates of individual-MIC were 8.0–21.1. For all scales but two, the individual-MIC values were larger than the group-MIC values.

**Conclusions:**

Interpretability of ThyPRO was improved by the establishment of MIC values, which was 6.3–14.3 for groups and 8.0–21.1 for individuals. Thus, estimates of which changes are clinically relevant, are now available for future studies. We recommend using MIC values found by ROC analyses to evaluate changes in groups of patients, whereas MIC values identified by a dual criterion, including the reliability of changes, should be used for individual patients, for example, to identify individual responders in clinical studies or practice.

## Introduction

During the last decades, the importance of assessing health-related quality of life (QoL) has been widely acknowledged, and QoL assessments are used both in comparative effectiveness research, and in patient-centered healthcare (1, 2, 3). The use of patient-reported outcomes measures as endpoints in clinical trials is now widespread on a par with traditional endpoints such as survival or tumor response in cancer (4, 5).

A general challenge for interpretation of patient-reported outcomes is to define whether an observed change in numerical scale score is large enough to be clinically relevant. Consequently, the term minimal important change (MIC) has been proposed (3). An MIC is defined as ‘the smallest change in score in the construct to be measured that patients perceive as important’ (6, 7, 8). The concept is similar to minimal clinical important difference (MCID) but emphasize change over time and the importance of patient perception. Establishing meaningful changes in patient-reported outcome ratings has been widely discussed over the last decades (9). Anchor- and distribution-based approaches are the main two methods to determine MICs (10). Anchor-based approaches apply an ‘anchor’ external to the instrument under evaluation. In QoL-research the anchor is often patient-rated. Distribution-based approaches utilize the statistical distributions of responses to the instrument under evaluation to establish MICs, for example, differences in central tendency measures in relation to variability measures. There is no consensus on the best way to determine the MIC, but anchor-based approaches are usually preferred, because the concept of minimal importance is based on patient assessment, whereas the distribution-based methods do not, in themselves, provide a good indication of the importance of the observed change from the patients’ perspective (10).

Anchor-based data can be analyzed by receiver operating characteristic (ROC) curve methods for estimation of MIC values, as recommended by Vet *et al*. (7). In classical clinical research applications of ROC analyses, a gold standard diagnostic test, constitutes an anchor, against which a new diagnostic test is assessed. In patient-reported outcome studies, a Global Rating of Change is applied as anchor, the Global Rating of Change being a rating scale designed to measure improvement/deterioration from the patient’s perspective (11).

The Reliable Change Index is traditionally included as one of the distribution-based methods to determine MIC (10). The Reliable Change Index is based on the standard error of measurement (s.e.m.) for the score of each patient, which is derived from the standard deviation and reliability. This method may be applied in order to support results from the ROC method, since the Reliable Change Index takes the measurement error of the change scores into account, which is not the case for the ROC method (10). The Reliable Change Index determines the limits for a change of the observed score for each patient if the true score is unchanged (12, 13).

Patients with benign thyroid diseases have higher morbidity and mortality than the general population (14, 15) and experience impaired quality of life (16, 17), often also when adequately treated (18, 19, 20), which calls for further patient-centered outcomes research (21, 22). Consequently, the ThyPRO questionnaire was developed and implemented as the international standard measure of thyroid-related QoL (23, 24). Its reliability, validity and responsiveness have been extensively documented (25, 26, 27, 28).

Reflecting the fact that various benign thyroid diseases are interrelated in etiology, symptomatology and through treatment, ThyPRO was intended to cover the whole spectrum of thyroid diagnoses. Therefore, MICs should be established in a cohort of patients covering the whole range of benign thyroid disorders.

The purpose of this study was to estimate MIC values for use of ThyPRO in groups as well as individual patients.

## Methods

### Study population

From 2008 to 2013, patients undergoing treatment for benign thyroid diseases at two university hospital outpatient clinics (Rigshospitalet and Odense University Hospital), were invited to complete ThyPRO prior to and 6 weeks after treatment initiation. At follow-up, patients also rated their change since baseline; both overall and for each of 13 specific domains measured by ThyPRO. Aiming at samples around 100 (based on previous experience) in each of the major thyroid disease groups, the inclusion criteria were: age above 18; ability to complete paper-and-pencil questionnaires in Danish; and referral to and prescription of clinically relevant treatment or change in treatment of thyroid disease. Exclusion criteria were: pregnancy; patients undergoing minor adjustments of treatment or referred for second opinion or diagnostic procedures; major comorbidity considered to have substantial influence on QoL; or thyroid malignancy. Eligible patients were identified through screening of all patients referred with a diagnosis of thyroid disease. Eligible patients received a booklet containing ThyPRO and sociodemographic questions by mail, followed by a reminder in case of nonresponse. Clinical data were obtained by medical chart review. A subset of the data has previously been used for a methodological evaluation of the responsiveness of the ThyPRO questionnaire (26).

### Patient-reported outcome (PRO) measures

The ThyPRO measures a range of aspects of QoL relevant to patients with benign thyroid disease. It covers not only physical symptoms specifically relevant to thyroid diseases, for example, symptoms of hyperthyroidism and goiter, but also nonspecific aspects of high importance to the patients, for example, depressive symptoms and impaired social life, identified by the patients themselves and clinical experts (29).

The ThyPRO consists of 85 items, summarized in 13 domain-specific multi-item scales, and one single-item overall QoL scale (Supplementary ThyPRO questionnaire (see section on supplementary materials given at the end of this article). Furthermore, a Composite QoL scale can be scored, by summarizing 22 items including the overall QoL item (30). Thus, MICs were established for 14 multi-item scales. Each individual item is rated on a 0–4 Likert scale (from no symptoms/problems to severe symptoms/problems). Scale scores are calculated as the simple sum of the items within the scale and transformed to yield score ranges from 0 to 100, with higher scores indicating more symptoms/problems.

### Global rating of change

At 6 weeks follow-up, patients were asked to rate their experienced change of each specific ThyPRO domain and their overall quality of life, after having responded to each of the corresponding ThyPRO scales. The Global Rating of Change questions were: ‘Compared to the last time you answered this questionnaire, do you feel that your *[relevant issue, e.g. tiredness]* all in all is better, worse or approximately the same?’. Patients rated their change on a 7-point Likert scale: a great deal worse, somewhat worse, a little worse, unchanged, a little better, somewhat better, a great deal better. The self-assessment value was used as the external anchor for defining MIC. Patients rating themselves a little better, somewhat better or a great deal better were considered importantly improved (31).

### Statistical analysis

The sensitivity of each ThyPRO scale was calculated as the proportion of importantly improved patients, according to the Global Rating of Change that were correctly identified as such, by the change in ThyPRO scale scores from baseline to follow-up (26). The specificity was calculated as the proportion of patients, correctly identified by ThyPRO, without an important improvement, according to the Global Rating of Change.

A ROC curve was produced for each ThyPRO scale by plotting the sensitivity against the 1 − specificity. The optimal ROC cut-off point was defined as the value for which the sum of the squared proportions of misclassifications ((1 − sensitivity)^2^ + (1 − specificity)^2^) was smallest. The change in scale score corresponding to the cut-off value was chosen as the group-level MIC. For simplicity, we report the absolute values for MIC, although a score improvement is indicated by a negative change score. To assess the strength of our findings, the area under the curve (AUC) of each ROC curve was calculated. In accordance with previous studies/recommendations (31), an AUC above 0.7 was considered acceptable, whereas an AUC greater than 0.8 is considered good, and an AUC greater than 0.9 represents excellent discrimination (31). An AUC of 0.5 means that the measure doesn’t discriminate better than chance (32).

The Reliable Change Index was calculated according to Jacobson *et al.* (12) and Liu *et al.* (13) using an 80% CI:


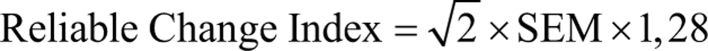


where


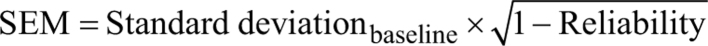


Reliability was estimated by Cronbach’s alpha (33).

All statistical analyses were performed using SAS Enterprise Guide Version 7.1.

### Ethical considerations

According to Danish law, PRO studies do not require and thus cannot obtain approval by ethical committees. A completed, returned survey is regarded as consent. The study was approved by the Danish Data Protection Agency (#2007-58-0015) and conducted in accordance with the Declarations of Helsinki.

## Results

Of the 779 patients invited to participate in the study, baseline evaluations were completed by 544 patients undergoing clinically relevant treatment, of whom 435 completed the follow-up survey, yielding a completion rate of 56% for the invited patients, and 80% for the patients completing baseline evaluations. Demographic and basic clinical characteristics are shown in Table 1. More detailed clinical description of the study population, has been provided in a previous clinical validation study (26).

**Table 1:** Clinical characteristics of the sample at baseline.

*n*	435
Gender	
Women	361 (83)
Men	74 (17)
Age (years)	54 (42–63)
Diagnosis	
Nontoxic goiter	135 (31)
Toxic nodular goiter	98 (23)
Graves’ hyperthyroidism	73 (17)
Graves’ orbitopathy (GO)	25 (6)
Autoimmune hypothyroidism	86 (20)
Other thyroid diagnoses	18 (4)
Disease duration (months)	0.3 (0–4)
Treatment instituted	
Levothyroxine	111 (26)
Antithyroid medication	86 (20)
Aspiration of thyroid cyst	4 (1)
Glucocorticoid pulse therapy of GO	2 (0)
Other immunosuppressive treatment of GO	4 (1)
Hemithyroidectomy	64 (15)
Total thyroidectomy	37 (9)
Radioactive iodine	127 (29)

Data are expressed as number (percentage) or median (interquartile range (Q1–Q3)).

At baseline, the mean scale scores ranged between 14 and 58, with the highest (i.e. worst) score on the Tiredness scale and the lowest score on the Impaired Social Life scale. For most of the remaining scales, mean baseline scores ranged between 20 and 30 points. The frequency of patients perceiving themselves as importantly improved were highest for the Goiter Symptoms scale with 45% perceiving themselves improved, and lowest for the Cosmetic Complaints and Impaired Sex Life scales with 13 and 14%, respectively. For the remaining scales, the frequency was between 21 and 39%. Mean change in scores varied between −1.1 for the depressivity scale and −12.0 for the Anxiety scale (Table 2; negative mean changes indicate improved quality of life).

**Table 2:** Results of the MIC analyses.

ThyPRO scale	Mean score at baseline (s.d.)	Mean change from baseline to follow-up	Smallest possible change^a^	AUC	ROC-based MIC	Sensitivity %	Specificity %	Cronbach’s alpha	sem	Reliable change index
Goiter symptoms	22 (21.6)	−8.1	2.27	**0.72**	**6.8**	69	71	0.92	6.2	**11.2**
Hyperthyroid symptoms	28 (27.7)	−9.9	3.13	**0.67**	**10.7**	55	72	0.84	8.6	**15.5**
Hypothyroid symptoms	24 (21.8)	−5.2	6.25	**0.64**	**6.3**	71	57	0.71	11.7	**21.1**
Eye symptoms	17 (19.1)	−4.1	3.13	**0.76**	**6.3**	68	73	0.86	7.0	**12.7**
Cognition	25 (23.9)	−5.4	4.17	**0.68**	**6.7**	63	69	0.94	6.0	**10.9**
Tiredness	58 (27.0)	−11.9	3.57	**0.85**	**14.3**	80	79	0.94	6.4	**11.6**
Anxiety	29 (24.1)	−12.0	4.17	**0.76**	**12.5**	75	69	0.91	7.4	**13.4**
Depressivity	33 (22.2)	−1.1	3.57	**0.77**	**7.1**	73	70	0.91	6.8	**12.3**
Emotional susceptibility	39 (24.6)	−9.4	2.78	**0.80**	**11.1**	72	72	0.94	6.3	**11.4**
Impaired daily life	25 (27.1)	−9.1	4.17	**0.80**	**7.5**	79	73	0.94	6.9	**12.5**
Impaired social life	14 (20.2)	−3.6	6.25	**0.71**	**6.3**	70	72	0.83	8.2	**14.9**
Impaired sex life	24 (30.9)	−6.6	12.50	**0.68**	**12.5**	66	72	0.90	9.8	**17.8**
Cosmetic complaints	19 (20.0)	−1.7	4.17	**0.65**	**8.3**	54	71	0.82	8.5	**15.3**
ThyPRO composite QoL	31 (15.8)	−1.7	1.14	**0.74**	**9.1**	68	75	0.95	4.4	**8.0**

^a^Smallest possible change in scale score for a single person.

AUC, area under the curve; MIC, minimal important change; ROC, receiver operating characteristics; Qol, quality of life.

### Group-level MIC – ROC curve analyses

The estimated MIC values are shown in Table 2 for each of the 13 ThyPRO multi-item scales and the composite QoL scale. The MIC values ranged between 6.3 (Hypothyroid Symptoms, Eye Symptoms and Impaired Social Life scales) and 14.3 (Tiredness scale). For the Hypothyroid Symptoms, Impaired Sex Life and Impaired Social Life scales, the estimated MIC was equal to the smallest possible improvement in scores for a single patient. For all other scales, the estimated MIC was larger than the smallest possible improvement. Nine of the 14 scale AUC values were above the recommended threshold of 0.7. The AUC’s of the remaining five scales were between 0.64 and 0.68, with the Hypothyroid Symptoms scale having the lowest AUC. The sensitivity was lowest for the Cosmetic Complaints and Hyperthyroid Symptoms scales, with a sensitivity of 54 and 55%, respectively, and highest for the Tiredness and Impaired Daily Life scales, with a sensitivity of 79%. For most of the other scales, sensitivity ranged between 65 and 75%. The specificity was lowest for the Hypothyroid Symptoms scale with a specificity of 56%, and highest for the Tiredness scale, with a specificity of 79%. For the rest of the scales, specificity ranged between 69 and 75%.

### Individual-level MIC – reliable change index

Except for the Tiredness and comosite QoL scales, Reliable Change Indices were higher than the anchor-based MIC values, as seen in Table 2. The Hypothyroid Symptoms scale had the highest Reliable Change Index of 21.1. For most of the other 12 scales with an Reliable Change Index greater than the anchor-based MIC values, the Reliable Change Index ranged between 10 and 14. Table 3 summarizes our recommendations regarding MIC for group differences and for intra-individual change. Table 4 shows the percentage of patients experiencing an individual-level MIC in each disease group.

**Table 3:** ThyPRO MIC levels recommended for future studies.

ThyPRO scale	Group-level MIC	Individual level/responder definition MIC
Goiter symptoms	6.8	11.2
Hyperthyroid symptoms	10.7	15.5
Hypothyroid symptoms	6.3	21.1
Eye symptoms	6.3	12.7
Cognition	6.7	10.9
Tiredness	14.3	14.3
Anxiety	12.5	13.4
Depressivity	7.1	12.3
Emotional susceptibility	11.1	11.4
Impaired daily life	7.5	12.5
Impaired social life	6.3	14.9
Impaired sex life	12.5	17.8
Cosmetic somplaints	8.3	15.3
ThyPRO composite QoL	9.1	9.1

MIC, Minimal Important Change.

**Table 4:** Percentage of patients in each diagnostic group reporting an improvement greater than the individual-level minimal important change, 6 weeks after treatment initiation.

ThyPRO scale	Non-toxic goiter	Toxic nodular goiter	Graves’ hyperthyroidism	Graves’ orbitopathy	Autoimmune hypothyroidism	Other thyroid diagnoses
Goiter symptoms	54	33	28	35	21	40
Hyperthyroid symptoms	25	23	63	53	21	36
Hypothyroid symptoms	11	16	25	14	13	7
Eye symptoms	15	26	17	35	13	27
Cognition	30	29	36	41	29	13
Tiredness	30	33	49	53	42	47
Anxiety	41	37	50	53	23	13
Depressivity	31	29	31	53	30	13
Emotional susceptibility	36	30	43	53	31	20
Impaired daily life	22	30	47	47	31	47
Impaired social life	14	14	17	35	19	13
Impaired sex life	20	25	28	20	34	36
Cosmetic complaints	15	11	17	13	13	7
ThyPRO composite QoL	40	35	49	53	36	27

## Discussion

The purpose of this study was to determine MIC values for the thyroid-related QoL questionnaire ThyPRO. We determined an MIC for each of the 13 multi-item scales of ThyPRO and for the Composite QoL scale, using an anchor-based method with a domain-specific Global Rating of Change as the anchor, as well as ROC curve analysis.

Using this approach, group-level MICs at levels comparable to those applied in previous research (22, 34), were established. These values may be used in classical power and sample size calculation for future clinical trials, comparing mean levels of groups. In some instances (e.g. important outcomes for non-toxic goiter interventions), smaller differences may be argued for (35). Another recommendable approach would be to estimate the proportion of treatment-responders, defined as patients experiencing improvement larger than the individual (Reliable Change Index-based) MIC levels in relevant groups, for example, intervention vs placebo group in RCTs. This may be particularly relevant if a treatment response is only expected in subgroups of patients (36).

The ROC curve analysis has previously been described as the point closest-to-(0, 1) corner in the ROC plane approach, and it has been shown to outperform other approaches (such as the Youden index) in identifying the best cut-off point (37). These analyses were supported by Reliable Change Index values for each scale. The Reliable Change Index was calculated according to Jacobson *et al.* (12) and Liu *et al.* (13) using an 80% CI. For group level results, a 95% CI is customary, and was used in the original Reliable Change Index paper (12). However, for assessing change of an individual patient, we believe that a 95% CI is too conservative and would lead to an unacceptable high misclassification of patients who had experienced a true change. For this reason, we chose a CI of 80%.

The MIC values were found to range between 6.3 and 14.3. For nine of the 14 scales, the association between the Global Rating of Change anchor and the change in score was of acceptable strength for MIC estimation, as the AUC’s were greater than 0.7. For the last five scales, the AUCs were between 0.64 and 0.68. Thus, to support the findings for these five scales, further studies need to be carried out. An MIC of half the size of the standard deviation at baseline has been suggested as a rule of thumb for an MIC (38). The anchor-based MIC estimates were generally smaller than half a standard deviation, while the Reliable Change Index estimates were of this magnitude or larger. For four scales (Tiredness, Anxiety, Emotional susceptibility and Impaired Sex Life), the MIC values calculated via the ROC method were large (more than 11 points). Three of these scales, Tiredness, Anxiety and Emotional Susceptibility, also showed a considerable mean improvement from baseline to follow-up. For the Impaired Sex Life scale, the high MIC was due to the smallest change in score being the same as the MIC, that is, the patient needs only to change one category on one item to be considered importantly changed.

The Reliable Change Indices were higher than the anchor-based MIC values in 12 of the 14 scales, with Reliable Change Indices between 11 and 21. Thus, by replacing the anchor-based MIC with the Reliable Change Index-based MIC when evaluating individual patients, risk of ‘false positive’ relevant changes (detailed discussion below) are minimized for these 12 scales.

Determining whether to use the anchor-based MIC or the MIC based on Reliable Change Index depends on the application of the MIC. If the MIC is to be used in studies looking at group differences, we recommend using the anchor-based MIC. The variability of the scores is minimized when evaluating group means, because the variability of the mean of a group is inversely proportional with the square root of the number of persons in the group. If, on the other hand, the study assesses patients individually, one should take the Reliable Change Index into account, since these results would be single measurements for each patient, and thus at risk of being influenced by random error. For this purpose, the Reliable Change Index value should be chosen as the MIC, if the Reliable Change Index is higher than the anchor-based MIC (which is the case for all but two scales). The same would apply to analyses evaluating proportions of treatment-responders in a group, rather than change in group means. The change in score for each of these responding individuals should exceed the highest value of anchor-based and Reliable Change Index-based MIC, to ensure that the changes in scores are reliable. This distinction between group evaluations and individual assessments has previously been described by Guyatt *et al.* (39), denominated ‘inferences concerning individuals and inferences concerning groups’. Guyatt *et al.* give an example of a small change in mean blood pressure (e.g. 2 mmHg) being of a magnitude that would be trivial for an individual, whereas a mean change of the same magnitude in a large population may translate into a large number of reduced strokes in that population (39). The two categories of MIC are presented in Table 3, Table 4 illustrates that the percentage of patients experiencing an individual-level MIC depends on both the diagnosis and outcome scale. For example, 30% of patients with non-toxic goiter experienced an improvement larger than the individual-level MIC, whereas this was about 50% in patients with Graves’ disease.

The MIC values can be used to compare treatment effects in longitudinal studies and for power calculations prior to future clinical trials. For example, in the sub-sample of patients with autoimmune hypothyroidism in the study by Winther *et al.* (17), the mean level of the Impaired Daily Life scale was improved from 22 to 14 after 6 weeks. Sample size calculations for an RCT attempting to improve that outcome further, would then be based on a decrement in mean score by MIC_g_ = 7.5, to 6.5 (corresponding to a total sample size around 410). Applying the alternative approach, based on individual responses, sample size calculations could be based on the proportion of responders presented in Table 4. For example, at 6 weeks, 31% of patients with autoimmune hypothyroidism had improved importantly (i.e. a change ≥ MIC_i_ =12.5) on the Impaired Daily Life scale. An RCT attempting to improve that proportion by for example, 30% should be dimensioned to identify a change to 40% (corresponding to a total sample size about 870).

A subset of data from the present study has previously been used to evaluate responsiveness for ThyPRO (26). In that study, clinicians determined which patient groups they anticipated would change in specific scales in 6 months. For the predefined patient groups expected to change, the mean changes in scale scores were larger than the present MIC values for all but two scales (Hypothyroid Symptoms and Cosmetic Complaints), indicating that the MIC values are in line with the expectations of the clinicians. Of all scales, the Hypothyroid Symptoms scale (which measures *physical* symptoms of hypothyroidism) had the lowest AUC, the highest Reliable Change Index and the lowest specificity. Additionally, the MIC value was equal to the smallest possible change in scale score. It has been suggested, that the physical symptoms assessed by the Hypothyroid Symptoms scale (primarily hair and skin changes) persist for a longer time than other symptoms, despite treatment (26). In the present study, patients with hypothyroidism on average experienced a change of 2 points on the Hypothyroid Symptoms scale, thus supporting this notion.

From a clinicians’ perspective, the categorization of patients applied here, and the lack of detailed clinical description of these, may seem odd and incomplete. Can patients with non-toxic goiter be grouped along with patients with Graves’ disease? And what were the clinical characteristics of the specific diagnostic groups? The point here is, that the relevant categorization is not a clinical one; the scope of this paper goes beyond clinical descriptions; the categorization is based on whether or not the patients have experienced an improvement, regardless of their particular thyroid diagnosis and treatment, in order to establish MICs for ThyPRO that is applicable across the classical clinical dividers. We chose to define the patients as having experienced an important improvement, if they reported a change of −1, −2 or −3 on the Global Rating of Change scale (a little better, somewhat better or a great deal better), since even a small change in only 6 weeks was considered important. It is a strength that a large group of patients with different benign thyroid diseases participated and completed follow-up. The 6 weeks follow-up was deliberately chosen in order for the patients to better remember their baseline status. In future studies, it would be interesting evaluate and compare with longer time periods, for example, 6 months follow-up.

In conclusion, we recommend employing the scale-specific MIC values for ThyPRO to assess change in quality of life in patients with thyroid disease. For group-level comparisons, we recommend that the anchor-based MIC values are chosen, whereas on the level of the individual, we recommend that the highest value of the Reliable Change Index and the anchor-based MIC are applied (for illustration and future application presented in a separate Table 3).

## Supplementary Material

Quality of Life Questionnaire

Scale content of the ThyPRO

## Declaration of interest

The authors declare that there is no conflict of interest that could be perceived as prejudicing the impartiality of the research reported.

## Funding

The project was supported by grants from the Mørk and the Wedell-Wedellsborg foundations, and the research salary of UFR was supported by a grant from NovoNordisk Foundation.

## Author contribution statement

Selma Flora Nordqvist: Data analyses, drafting and approving the final manuscript; Victor Brun Boesen: Data analyses, drafting and approving final manuscript; Åse Krogh Rasmussen: Study design, data acquisition, revising and approving final manuscript; Ulla Feldt-Rasmussen: Study design, data acquisition, revising and approving final manuscript; Laszlo Hegedüs: Study design, data acquisition, revising and approving final manuscript; Steen Joop Bonnema: Study design, data acquisition, revising and approving final manuscript; Per Karkov Cramon: Data acquisition, analyses, drafting and approving final manuscript; Torquil Watt: Study design, data acquisition, revising and approving final manuscript; Mogens Groenvold: Study design, revising and approving final manuscript; Jakob Bue Bjorner: Study design, data analyses, drafting and approving the final manuscript.
