# Supplementary material for: Determining minimal important change for the thyroid-related quality of life questionnaire ThyPRO
Source: Endocr Connect. 2021 Feb 10;10(3):316–24. doi: 10.1530/EC-21-0026 (PMC8052573; doi:10.1530/EC-21-0026)
Supplement: Scale content of the ThyPRO [file supplementary_table_2.pdf]

Scale content of the ThyPRO

The ThyPRO scales consist of the following items, summarized within each scale to form a scale score for each scale ranging 0-100.

Goitre symptoms

During the past 4 weeks have you

1a	- had the sensation of fullness in the neck?
1b	- had a visible swelling in the front of your neck?
1c	- felt pressure in your throat?
1d	- had pain in the front of your throat?
1e	- had pain in your neck that could be felt in your ears?
1f	- had the sensation of a lump in your throat?
1g	- had the need to clear your throat frequently?
1h	- felt discomfort swallowing?
1i	- had difficulty swallowing?
1j	- had the sensation of suffocating?
1k	- been hoarse?

Hyperthyroid symptoms:

During the past 4 weeks have you

1l	- had trembling hands?
1m	- had a tendency to sweat a lot?
1n	- experienced palpitations (rapid heart beat)?
1o	- experienced shortness of breath?
1p	- been sensitive to heat?
1r	- had an increased appetite?
1s	- had loose stools?
1t	- had an upset stomach?

Hypothyroid symptoms:

During the past 4 weeks have you

1q	- been sensitive to cold?
1cc	- had swollen hands or feet?
1dd	- had dry skin?
1ee	- had itchy skin?

Eye symptoms:

During the past 4 weeks have you

1u	- had moist or watery eyes?
1v	- had bags under the eyes or swollen eyelids?
1w	- had the sensation of dryness or “grittiness” in the eyes?
1x	- had impaired vision?
1y	- felt pressure in (or behind) the eyes?
1z	- had double vision?
1aa	- had eye pain?
1bb	- been very sensitive to light?

Tiredness:

During the past 4 weeks have you

2a	- been tired?
2b	- been exhausted?
2c	- had difficulty getting motivated to do anything at all?
2d	- felt worn out?
3a	- felt full of life?*
3b	- felt energetic?*
3c	- been able to cope with the demands of your life?*

Cognitive problems:

During the past 4 weeks have you

4a	- had difficulty remembering?
4b	- had slow or unclear thinking?
4c	- had difficulty finding the right words?
4d	- been confused?
4e	- had difficulty learning something new?
4f	- had difficulty concentrating?

Anxiety:

During the past 4 weeks have you

5a - felt nervous?
5b - felt afraid or anxious?
5c - felt tense?
5d - been concerned about being seriously ill?
5e - felt uneasy?
5f - felt restless?

Depressivity:

During the past 4 weeks have you

6a - felt sad?
6b - felt depressed?
6c - felt discouraged?
6d - cried easily?
6e - felt unhappy?
6f - felt happy?
6g - had self-confidence?

Emotional Susceptibility:

During the past 4 weeks have you

7a - had difficulty coping?
7b - felt "not like yourself"?
7c - noticed you easily felt stressed?
7d - had mood swings?
7e - felt irritable?
7f - felt frustrated?
7g - felt angry?
7h - felt in control of your life?*
7i - felt in balance?*

Impaired Social life:

During the past 4 weeks, has your thyroid disease caused you to

8a - have difficulty being together with other people (for example, spouse, children, boy/girlfriend, friends, or others)?

8b - feel you were a burden to other people?

8c - have conflicts with other people?

During the past 4 weeks have you

8d - felt that people in your surroundings have lacked understanding of your thyroid disease?

Impaired Daily life:

During the past 4 weeks, has your thyroid disease caused you to

9a - have difficulty managing your daily life?

9b - limit your leisure activities or hobbies?

9c - not be able to participate in life around you?

9d - have difficulty getting around (for example, walking, running, bicycling, or driving a car)?

9e - feel as if everything takes longer to do?

9f - have difficulty managing your job (for example, finding it hard to cope or calling in sick)

Impaired Sexlife:

During the past 4 weeks have you

10a - felt your thyroid disease had a negative influence on your sex life?

10b - had a decreased sexual desire?

Cosmetic Complaints:

During the past 4 weeks

11a - has your thyroid disease affected your appearance (for example, swelling of the neck, eye changes, weight changes)?

11b - have you been unsatisfied with your appearance because of your thyroid disease?

11c - have you tried to camouflage or mask visible signs of your thyroid disease (for example, by wearing a scarf or sunglasses)?

11d - have you been bothered by other people looking at you?

11e - has your thyroid disease influenced which clothes you wear?

11f - has your thyroid disease made you feel too fat?

Overall Quality of life impact:

During the past 4 weeks

12 - has your thyroid disease had a negative effect on your quality of life?

Composite scale:

The Composite scale is based on the 22 items from the Tiredness, Cognition, Anxiety, Depressivity, Emotional Susceptibility, Impaired Social life Impaired Daily Life and Overall QoL-impact scales: 2a, 2c, 3b*, 4a, 4b, 4f, 5b, 5c, 5e, 6a, 6e, 6g*, 7c, 7d, 7h*, 8a, 8b, 8c, 9a, 9c, 9e, 12

*Positively worded items are scored reversely when constructing scales
